# Bone mineral density and trabecular bone score in Chinese subjects with sarcopenia

**DOI:** 10.1007/s40520-019-01266-8

**Published:** 2019-07-17

**Authors:** Hanmei Qi, Yunlu Sheng, Shu Chen, Siting Wang, Aisen Zhang, Jinmei Cai, Bing Lai, Guoxian Ding

**Affiliations:** 1 Division of Geriatric Endocrinology, The First Affiliated Hospital of Nanjing Medical University, 300 Guangzhou Road, Nanjing, 210029 Jiangsu China; 2 Division of Intensive Care Unit, Nanjing Jiangning Hospital, Nanjing, Jiangsu China

**Keywords:** Sarcopenia, Osteoporosis, Bone mineral density, Trabecular bone score

## Abstract

**Background:**

As the general population is aging worldwide, the incidence of sarcopenia and osteoporosis is also rapidly increasing. Studies have found the link between sarcopenia and osteoporosis, but the relationship between sarcopenia and osteoporosis, especially bone microarchitecture, remains unclear.

**Aims:**

To investigate the relationship between components of sarcopenia (muscle mass, handgrip strength, and gait speed) and components of osteoporosis [bone mass measured by bone mineral density (BMD) and bone microarchitecture measured by trabecular bone score (TBS)] in Chinese subjects.

**Methods:**

318 Chinese men and 203 Chinese women were included in our study. Muscle mass and BMD were measured by dual-energy X-ray absorptiometry (DXA). TBS iNsight^®^ software was used for TBS. Jamar hydraulic hand dynamometer was used to assess muscle strength, and gait speed was used to assess physical performance.

**Results:**

We found that the relative appendicular skeletal muscle mass (RASM) in both genders and handgrip strength in women correlated positively with TBS, RASM in men and handgrip strength in women correlated positively with BMDs. In the multiple linear regression model, RASM was positively associated with TBS in both genders, but no significant association was observed between RASM and BMDs. Interestingly, handgrip strength showed positive association with all evaluated BMDs and TBS in women, but not in men. Women with sarcopenia had lower TBS and BMDs at all evaluated sites. Men with sarcopenia had lower BMDs only at femur neck and total hip.

**Conclusions:**

The reduction of muscle mass and strength was significantly associated with decreased bone mass and deteriorated bone microarchitecture. More importantly, low muscle mass is an independent risk factor for bone microarchitecture in Chinese subjects.

## Introduction

Aging is related to numerous structural and functional changes that can result in frailty, disability, and death. The typical features of aging in the musculoskeletal system are a gradual loss of bone mass and deterioration of bone microarchitecture, progressive decline in muscle mass, strength, and function, leading to osteoporosis and sarcopenia [1].

As the general population is aging worldwide, the incidence of osteoporosis and sarcopenia is also rapidly increasing. A 2010 survey in the USA revealed that in people aged 50 years and older, about 53.6 million had osteoporosis or low bone mass, accounting for 54% of this population [2]. In China, the incidence of osteoporosis is over 40% in women and 25% in men aged over 50 years, and about 64.6% of women and 57.6% of men aged over 50 years have osteopenia [3]. Globally, as many as 50 million people are estimated to have sarcopenia [4]. Osteoporosis and sarcopenia can cause falls and fractures, and are the major causes for disability and death in the elderly population. More than 20% of the patients aged ≥ 50 years die within the first year after hip fracture, while around 50% of the survivors have disability, bringing heavy burdens to the society and families [5].

Muscles and bones are closely related, since they are physically adjacent to each other and commonly regulated by multiple elements. The muscle can exert influence on the bone via the neuroendocrine system and mechanical forces, but the exact effect is not fully understood [6, 7]. Many studies have evidenced the link between sarcopenia and osteoporosis, but the results are inconsistent due to current divergent diagnostic criteria and evaluation methods for sarcopenia and osteoporosis. The majority of studies have focused on the relationship between muscle mass and BMD, while only a few have assessed the effect of sarcopenia on bone quality [8–11]. However, about two-thirds of fracture patients do not have osteoporosis as defined by BMD values [12]. Type 2 diabetes patients have higher BMD than the healthy controls, but the risk of fracture increases [13]. These results suggested that the BMD alone is insufficient to evaluate bone strength and estimate the risk for fracture.

TBS is a novel and non-invasive imaging technique to measure the bone microarchitecture based on 3D bone characteristics (e.g., the trabecular number, trabecular separation, and connectivity density). Many studies indicated that TBS had a close association with bone quality, and supported the predictive ability of TBS for fracture risk independent of BMD [14, 15].

This study aimed to investigate the relationship between components of sarcopenia (muscle mass, handgrip strength, and gait speed) and components of osteoporosis (bone mass measured by BMD and bone microarchitecture measured by TBS) in Chinese subjects.

## Materials and methods

### Participants

The study recruited 506 Chinese men and 362 Chinese women who had the annual health examination at our hospital between June 2013 and October 2017. 139 male and 110 female subjects who had suffered from a disease that has potential impact on bones and/or soft tissues (such as thyroid dysfunction, rheumatoid arthritis, inflammatory myopathy, Parkinson’s disease, stroke, myocardial infarction, severe liver disease, creatinine clearance < 30 ml/min, or cancer) or used medication in the past 6 months (such as bisphosphonate, parathyroid hormone, estrogens, and glucocorticoids) were excluded. Subjects (17 men and 14 women) who had received spine surgery as well as those (18 men and 20 women) with a ≥ 35 kg/m^2^ or < 18.5 kg/m^2^ body mass index (BMI) were also excluded. Then, subjects (14 men and 15 women) who were unable to walk independently or had severe painful hand or wrist problems were excluded. Finally, 318 men (age range 33–92 years) and 203 women (age range 41–90 years) were included in this study.

The characteristics of the subjects [age, height, body weight, waist circumference, hip circumference, smoking, drinking, age of menopause, common co-morbidities such as hypertension, and type 2 diabetes mellitus (T2DM)] were recorded. The levels of fasting blood glucose, glycated hemoglobin, triglycerides, total cholesterol, and high-density and low-density lipoprotein cholesterol were also examined. Smoking was classified as current smoker (more than one cigarette per day in the last year) or non-smoker, and drinking was presented as yes when the participant’s alcohol intake was ≥ 10 g/week.

### Measurement of BMD and body composition

BMDs (g/cm^2^) at lumbar spine (LS), femoral neck (FN) and total hip (TH), total fat mass (FM), total lean mass (LM), and limb lean mass were measured by a DXA scanner (Hologic Inc, Bedford, MA, USA). BMD for spine represented the BMD of L1–L4 combined. Appendicular lean mass (aLM) was calculated as the sum of lean mass in the four limbs. As absolute muscle mass correlates with height, RASM was determined as aLM divided by height squared (aLM/height^2^, kg/m^2^). Percentage fat mass (PFM) was calculated as the ratio of total body FM divided by weight.

### Measurement of TBS

TBS measurements were conducted using TBS iNsight^®^ software (Version 2.0.0.1, Med-Imaps, Bordeaux, France) based on lumbar spine DXA files. The software used the anteroposterior spine raw DXA image in the same region as the BMD measurement. Anthropomorphic phantoms were used to calibrate the instruments.

### Muscle strength and gait speed assessment

Muscle strength was evaluated by the hydraulic hand dynamometer (Jamar^®^, Los Angeles, CA, USA). The subject exerted a maximum manual pressure with the dominant hand in the sitting position. Three attempts were performed and recorded with a 1-min interval; the highest value (in kilograms) was taken for analysis.

Gait speed was measured by a timed 4-m walk at usual paces as the average of two attempts. A slower than 0.8-m/s speed was considered as slow walking.

A single examiner conducted the evaluations independently in a silent room to avoid distractions during the procedures.

### Diagnosis of sarcopenia

According to the European Working Group on Sarcopenia in Older People (EWGSOP), sarcopenia was sub-classified into three conceptual stages: pre-sarcopenia (low muscle mass only); sarcopenia (low muscle mass + low muscle strength/low physical performance); severe sarcopenia (low muscle mass + low muscle strength + low physical performance) [4]. Severe sarcopenia was not measured in this study.

RASM below a threshold of 7.26 kg/m^2^ (male) or 5.5 kg/m^2^ (female) was defined as low muscle mass. Handgrip strength < 30 kg (male) or < 20 kg (female) was defined as low muscle strength. Gait speed slower than 0.8 m/s was defined as low physical performance.

### Statistical analysis

Data of the subjects’ characteristics were presented as the mean ± standard deviation or *n* (%). Differences in basic characteristics by gender were compared using Student’s *t* test for continuous variables and Chi-square test for categorical variables. One-way ANOVA with Tukey’s post hoc analysis was performed to detect the difference of BMD and TBS among the normal, pre-sarcopenia, and sarcopenia groups. Pearson’s correlation analysis was used to determine the association between body composition, handgrip strength, and BMDs or TBS. Multiple linear regression model was further applied for TBS and BMD analyses with age, BMI, smoking, alcohol drinking, hypertension, and T2DM adjusted. All statistical analyses were performed with SPSS 20.0 (IBM Corp, Armonk, NY, USA), and a statistical significance was defined as *p* < 0.05.

## Results

### Descriptive statistics

Table 1 shows the characteristics of subjects (318 men and 203 women). As expected, men had lower absolute and relative FM, and higher LM, RASM, and handgrip strength than women. Their regional BMDs and TBS were also higher than women. Considering these differences, we performed further analyses separately based on genders.

**Table 1:** Comparison of anthropometrics, clinical parameters, body composition, handgrip strength, BMDs, and TBS of study participants by gender

Characteristic	Male (*n* = 318)	Female (*n* = 203)	*p*
Age (year)	64.8 ± 12.9	67.4 ± 8.2	0.005
Height (cm)	170.3 ± 5.8	156.4 ± 5.8	< 0.001
Weight (kg)	72.8 ± 9.7	59.3 ± 8.0	< 0.001
BMI (kg/m^2^)	25.1 ± 2.8	24.2 ± 2.9	0.001
Waist circumference (cm)	91.0 ± 8.3	84.7 ± 8.8	< 0.001
Hip circumference (cm)	96.8 ± 6.0	95.4 ± 5.7	0.062
Waist-to-hip ratio	0.9 ± 0.1	0.9 ± 0.1	< 0.001
FBG (mmol/L)	6.2 ± 2.0	5.6 ± 1.5	< 0.001
HbA1c (%)	6.5 ± 1.3	6.2 ± 0.8	0.035
TG (mmol/L)	1.8 ± 1.4	1.7 ± 1.1	0.335
TC (mmol/L)	4.6 ± 1.1	5.0 ± 1.1	< 0.001
HDL-C (mmol/L)	1.1 ± 0.3	1.4 ± 0.4	< 0.001
LDL-C (mmol/L)	2.9 ± 0.8	2.9 ± 0.9	0.903
Age of menopause	–	49.9 ± 3.9	–
Current smoker (%)	34.0	0.5	< 0.001
Alcohol use (%)	37.4	4.4	< 0.001
Hypertension (%)	54.7	55.9	0.784
T2DM (%)	42.8	23.8	< 0.001
FM (kg)	20.65 ± 4.65	23.27 ± 5.16	< 0.001
PFM (%)	0.28 ± 0.04	0.39 ± 0.05	< 0.001
LM (kg)	48.58 ± 6.16	33.95 ± 4.36	< 0.001
aLM (kg)	20.80 ± 2.93	13.71 ± 1.99	< 0.001
RASM (kg/m^2^)	7.16 ± 0.82	5.59 ± 0.72	< 0.001
Handgrip strength (kg)	37.75 ± 8.18	24.03 ± 4.16	< 0.001
BMD (g/cm^2^)			
Femoral neck	0.766 ± 0.120	0.644 ± 0.115	< 0.001
Total hip	0.924 ± 0.137	0.781 ± 0.120	< 0.001
Lumbar spine	1.010 ± 0.154	0.829 ± 0.138	< 0.001
TBS	1.241 ± 0.105	1.129 ± 0.116	< 0.001

Variables are expressed as mean ± SD. *p* values were determined using Student’s *t* test for continuous variables and Chi-square test for categorical variables

*BMI* body mass index, *FBG* fasting blood glucose, *HbA1c* glycated hemoglobin, *TG* triglycerides, *TC* total cholesterol, *HDL-C* high-density lipoprotein cholesterol, *LDL-C* low-density lipoprotein cholesterol, *T2DM* type 2 diabetes mellitus, *FM* total fat mass, *PFM* percentage fat mass, *LM* total lean mass, *aLM* appendicular lean mass, *RASM* relative appendicular skeletal muscle mass, *BMD* bone mineral density, *TBS* trabecular bone score

### Correlation between body composition, handgrip strength, and BMDs or TBS

Table 2 presents the unadjusted and adjusted correlation coefficients between body composition, handgrip strength, and BMDs or TBS. In men, after adjusting age and BMI, both BMDs and TBS were correlated negatively with absolute and relative FM, but positively with muscle mass including LM, aLM, and RASM. There was no significant correlation between handgrip strength and BMDs or TBS. In women, lean mass and handgrip strength correlated positively with both BMDs and TBS, while TBS correlated negatively with absolute and relative FM.

**Table 2:** Correlation between body composition, handgrip strength, and BMDs or TBS

	*r*				Age and BMI adjusted *r*			
	BMD			TBS	BMD			TBS
	FN	TH	LS		FN	TH	LS	
Male								
FM	0.177^**^	0.252^***^	0.110^*^	− 0.429^***^	− 0.11^*^	− 0.063	− 0.117^*^	− 0.301^***^
PFM	− 0.02	0.096	0.038	− 0.424^***^	− 0.149^**^	− 0.064	− 0.141^*^	− 0.299^***^
LM	0.367^***^	0.306^***^	0.159^**^	− 0.094	0.173^**^	0.053	0.132^*^	0.121^*^
aLM	0.334^***^	0.269^***^	0.100	− 0.043	0.134^*^	0.028	0.072	0.142^*^
RASM	0.317^***^	0.289^***^	0.114^*^	− 0.067	0.213^**^	0.223^**^	0.216^**^	0.232^**^
Handgrip strength	0.142^*^	0.111^*^	− 0.028	0.06	0.045	0.047	0.053	0.023
Female								
FM	0.313^***^	0.302^***^	0.299^***^	− 0.399^***^	0.104	0.071	0.017	− 0.456^***^
PFM	0.080	0.097	0.080	− 0.548^***^	− 0.043	− 0.027	− 0.076	− 0.538^***^
LM	0.436^***^	0.375^***^	0.355^***^	0.230^**^	0.260^***^	0.167^*^	0.137	0.375^***^
aLM	0.374^***^	0.325^***^	0.291^***^	0.298^***^	0.192^**^	0.123	0.086	0.402^***^
RASM	0.217^**^	0.214^**^	0.215^**^	0.235^**^	0.026	0.015	− 0.004	0.427^***^
Handgrip strength	0.445^***^	0.401^***^	0.375^***^	0.265^***^	0.349^***^	0.299^***^	0.304^***^	0.196^**^

*r* correlation coefficient

*BMD* bone mineral density, *TBS* trabecular bone score, *FN* femoral neck, *TH* total hip, *LS* lumbar spine, *FM* total fat mass, *PFM* percentage fat mass, *LM* total lean mass, *aLM* appendicular lean mass, *RASM* relative appendicular skeletal muscle mass

*p < 0.05; **p < 0.01; ***p < 0.001

### Multiple regression analysis of RASM, handgrip strength and BMDs or TBS

In the multiple linear regression model adjusted for age, BMI, hypertension, T2DM, the smoking status, and the alcohol drinking (Table 3), RASM was positively correlated with TBS in both genders (*β* = 0.306, *p* = 0.001 in men, *β* = 0.469, *p *< 0.001 in women). No significant correlation was found between RASM and BMDs. Interestingly, handgrip strength showed positive association with all evaluated BMDs and TBS in women, but not in men.

**Table 3:** Multiple linear regression analyses of RASM and handgrip strength on BMDs and TBS

Variables	Male			Female		
	Standardized *β*	*t*	*p*	Standardized *β*	*t*	*p*
FN BMD						
Age	− 0.160	− 2.322	0.021	− 0.354	− 5.403	< 0.001
BMI	0.221	2.589	0.010	0.271	3.726	< 0.001
Current smoker	− 0.167	− 2.794	0.006	0.065	1.047	0.296
Alcohol use	− 0.002	− 0.041	0.967	− 0.019	− 0.311	0.756
Hypertension	− 0.153	− 2.788	0.006	0.018	0.273	0.786
T2DM	0.139	2.471	0.014	− 0.013	− 0.208	0.836
RASM	0.078	0.846	0.398	− 0.014	− 0.192	0.848
Handgrip strength	0.034	0.509	0.611	0.291	4.625	< 0.001
	*R*^2^ = 0.159			*R*^2^ = 0.340		
TH BMD						
Age	− 0.067	− 0.964	0.336	− 0.320	− 4.754	< 0.001
BMI	0.362	4.178	< 0.001	0.294	3.932	< 0.001
Current smoker	− 0.112	− 1.854	0.065	0.070	1.092	0.276
Alcohol use	− 0.008	− 0.136	0.892	− 0.003	− 0.047	0.963
Hypertension	− 0.098	− 1.759	0.080	− 0.018	− 0.275	0.784
T2DM	0.116	2.032	0.043	0.013	0.208	0.835
RASM	− 0.050	− 0.534	0.594	− 0.027	− 0.370	0.712
Handgrip strength	0.066	0.983	0.326	0.270	4.170	< 0.001
	*R*^2^ = 0.133			*R*^2^ = 0.302		
LS BMD						
Age	0.170	2.369	0.018	− 0.221	− 3.172	0.002
BMI	0.203	2.277	0.024	0.327	4.221	< 0.001
Current smoker	− 0.134	− 2.152	0.032	− 0.020	− 0.294	0.769
Alcohol use	− 0.056	− 0.933	0.352	0.041	0.621	0.536
Hypertension	− 0.058	− 1.010	0.313	− 0.015	− 0.211	0.833
T2DM	0.138	2.348	0.020	0.041	0.642	0.522
RASM	− 0.002	− 0.021	0.983	− 0.038	− 0.497	0.620
Handgrip strength	0.081	1.169	0.243	0.272	4.047	< 0.001
	*R*^2^ = 0.086			*R*^2^ = 0.250		
TBS						
Age	− 0.191	− 2.877	0.004	− 0.230	− 3.431	0.001
BMI	− 0.572	− 6.942	< 0.001	− 0.473	− 6.363	< 0.001
Current smoker	− 0.188	− 3.269	0.001	− 0.044	− 0.690	0.491
Alcohol use	− 0.016	− 0.286	0.775	− 0.034	− 0.540	0.590
Hypertension	− 0.173	− 3.266	0.001	− 0.061	− 0.930	0.353
T2DM	0.161	2.949	0.003	− 0.013	− 0.209	0.835
RASM	0.306	3.444	0.001	0.469	6.452	< 0.001
Handgrip strength	− 0.052	− 0.812	0.417	0.135	2.099	0.037
	*R*^2^ = 0.214			*R*^2^ = 0.310		

Adjusted for age, BMI, smoking, alcohol drinking, hypertension, and T2DM

*β* standardized coefficient

*FN BMD* femoral neck bone mineral density, *TH BMD* total hip bone mineral density, *LS BMD* lumbar spine bone mineral density, *TBS* trabecular bone score, *BMI* body mass index, *T2DM* type 2 diabetes mellitus, *RASM* relative appendicular skeletal muscle mass

### Effect of sarcopenia status on BMDs and TBS

**Fig. 1:**
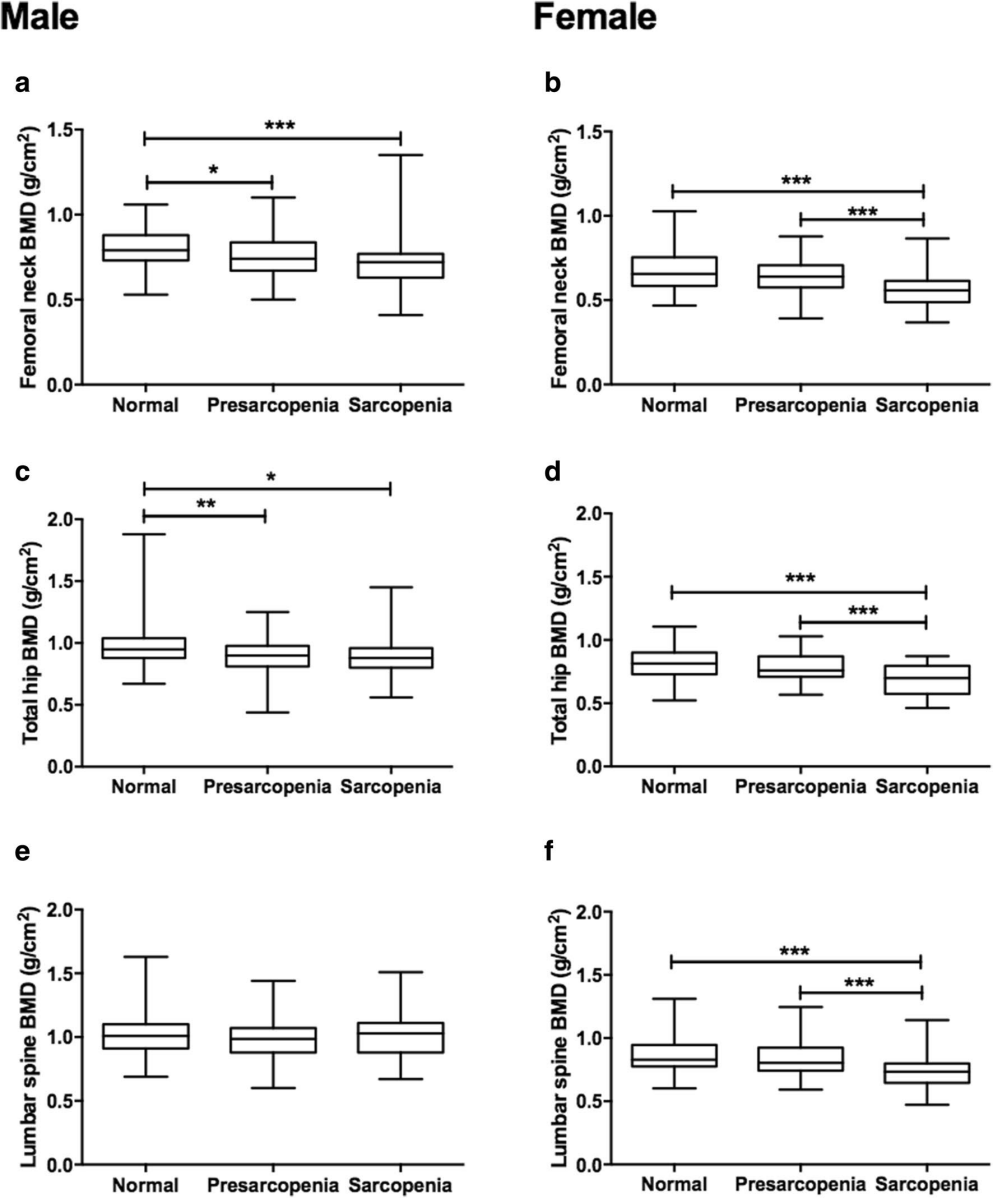
BMDs in different stages of sarcopenia. Femoral neck BMD (**a**, **b**), Total hip BMD (**c**, **d**), and Lumbar spine BMD (**e**, **f**) in normal, pre-sarcopenia, and sarcopenia men and women, respectively. Sarcopenia was judged using the definition of EWGSOP. One-way ANOVA with Tukey’s post hoc analysis was used among the three groups. *p < 0.05; **p < 0.01; ***p < 0.001. *BMD* bone mineral density

**Fig. 2:**
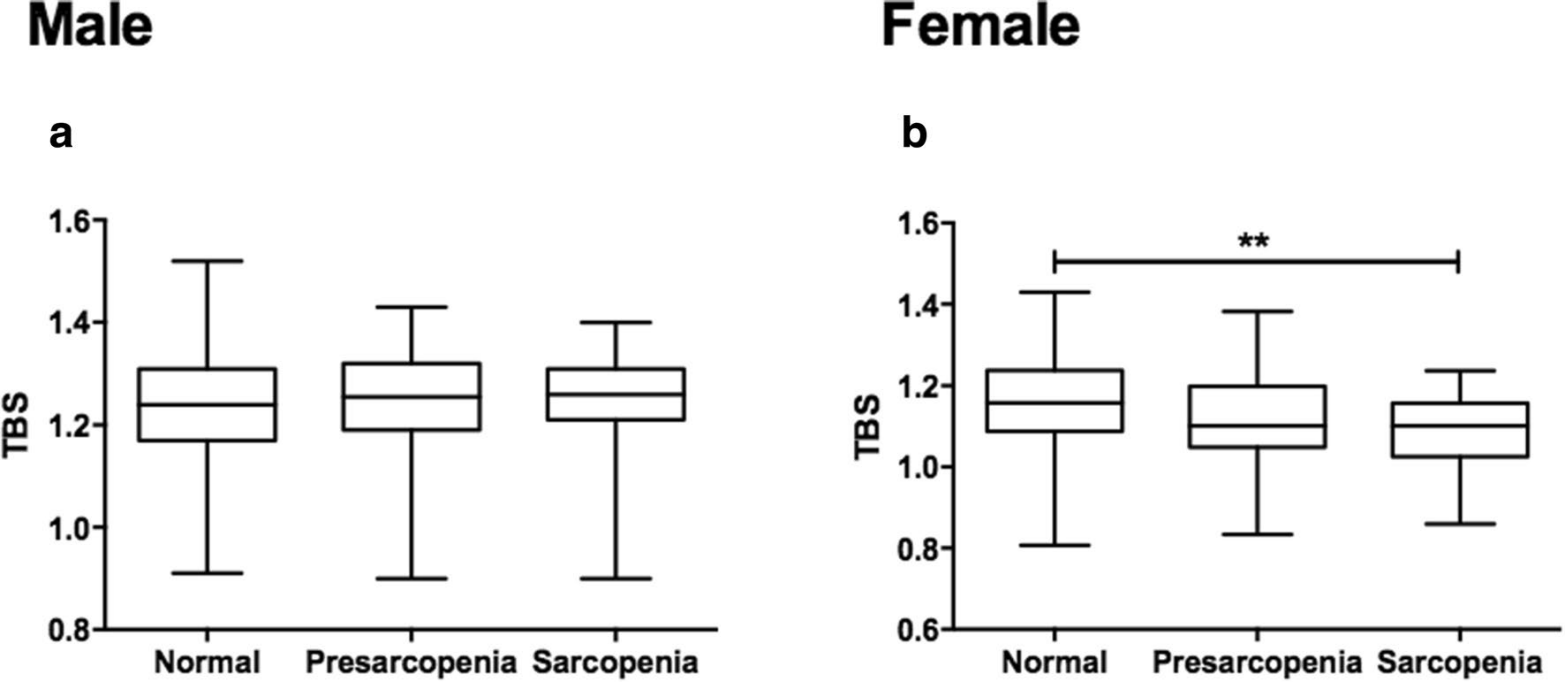
TBS in different stages of sarcopenia. TBS (**a**, **b**) in normal, pre-sarcopenia, and sarcopenia men and women, respectively. Sarcopenia was judged using the definition of EWGSOP. One-way ANOVA with Tukey’s post hoc analysis was used among the three groups, **p < 0.01. *TBS* trabecular bone score

Given the significant relationship between muscle and BMD and bone quality, we further performed subgroup analyses by stages of sarcopenia such as pre-sarcopenia and sarcopenia. Women with sarcopenia had lower TBS and BMDs at all the evaluated sites. Men with sarcopenia also had lower BMDs, but only at femur neck and total hip. The lumbar spine BMD and TBS were not significantly different among men with or without sarcopenia. The results are shown in Figs. 1 and 2.

## Discussion

Numerous studies have revealed a close functional and developmental relationship between muscle and bone [6, 7]. Genetic, endocrine, and mechanical factors as well as inflammatory and nutritional status impact both muscle and bone metabolism. However, the interactions between muscle and bone remain unclear [8–11].

More and more studies have found that the incidence of fractures significantly increases in patients with sarcopenia. In a prospective study of 2941 elderly subjects with a 6.6-year follow-up, an increased risk of hip fracture was found in sarcopenia patients [16]. Another prospective study in Hong Kong, China reported 11.3-year follow-up of elderly men, and revealed sarcopenia as an independent risk factor for bone fracture, apart from BMD and other risk factors [17]. Nonetheless, results from these studies were inconsistent due to different test strategies and reference populations, as well as varied diagnostic criteria and evaluation methods for sarcopenia and osteoporosis. Therefore, the relationship between sarcopenia and osteoporosis needs further study.

Our study analyzed the association between muscle mass and BMD using RASM, a widely used indicator for skeletal muscle mass. After adjustment for age and BMI, we found a positive association between RASM and BMDs at all evaluated sites in men. Nevertheless, in the multiple linear regression model adjusted for age, BMI, hypertension, T2DM, the smoking status, and the alcohol drinking, no significant correlation was found between RASM and BMDs in both genders.

However, apart from muscle mass decrease, the reduction of muscle strength is another main feature of sarcopenia. Hence, we further probed the relationship between handgrip strength and BMD. Multiple linear regression analysis revealed a positive association between handgrip strength and all evaluated BMDs in women, but not in men. The previous studies showed inconsistent results regarding the association of handgrip strength and BMD. A study of 234 men (mean age 47.8) showed that handgrip strength was not a good BMD predictor [18]. In contrast, Pereira and colleagues found that handgrip strength was positive associated with BMD in elderly men [19]. Our study suggests that handgrip strength has more influence on women’s BMD compared with men.

Besides BMD, bone strength, and fracture risk are also determined by bone quality. TBS, a new and non-invasive imaging technique, measures the bone microarchitecture based on 3D bone characteristics like the number and separation of trabecular, and connectivity density. The increased TBS indicates that the bone is strong with a lower risk for fracture. Many published studies showed that TBS was powerful in assessing bone quality, and supported the predictive ability of TBS for fracture risk. In addition, TBS is not influenced by osteoarthritis of lumbar spine [14, 15].

Only a few investigations have assessed the relationship between sarcopenia and bone quality. However, these studies used invasive techniques like peripheral CT to assess bone quality, and thus limited its application in humans [20, 21]. TBS is a non-invasive technique to assess bone quality in humans; hence, the relationship between sarcopenia and TBS is more meaningful.

As far as we know, the present study is the first to investigate the association between components of sarcopenia and TBS in Asians. The results indicated a positive correlation between muscle mass and TBS in both genders, while handgrip strength was only positively correlated with women’s TBS. In addition, women with sarcopenia had lower TBS. Our results differed from the study by Locquet group on European Caucasians, which showed that TBS had no significant reduction in sarcopenic patients of both genders [22]. These inconsistent results might due to different races of the study population and varied evaluation methods.

Furthermore, after controlling for potential confounders (age, BMI, co-morbidities, smoking status, and alcohol drinking status), RASM was positively correlated with TBS in both genders but not with BMDs. Our findings suggest that the decrease of muscle mass may deteriorate bone microarchitecture rather than bone mass, and low muscle mass is an independent risk factor for bone microarchitecture. However, there also existed some limitations in our study. First, since it was a cross-sectional study, we did not establish a cause-and-effect relationship between sarcopenia and osteoporosis. Second, as a clinical study, the mechanism of bone mass and quality reduction in sarcopenia patients could not be explained. Moreover, this was a single-centered study.

## Conclusions

The present study served as the first clinical evidence among Chinese subjects to show that the reduction of muscle mass and strength was significantly associated with decreased bone mass and deteriorated bone microarchitecture. More importantly, low muscle mass is an independent risk factor for bone microarchitecture. Therefore, early prophylactic measures for sarcopenia are probably critical for preventing osteoporotic fractures.
